# Acquired thrombotic thrombocytopenia purpura associated with severe ADAMTS13 deficiency in a 3-year-old boy: a case report and review of the literature

**DOI:** 10.1186/s13256-018-1806-9

**Published:** 2018-09-17

**Authors:** Hamidah Alias, Woon Lee Yong, Farah Azima Abdul Muttlib, Ho Wai Koo, C-Khai Loh, Sie Chong Doris Lau, Hafiza Alauddin, Raja Zahratul Azma

**Affiliations:** 1 Department of Pediatrics, UKM Medical Centre, Faculty of Medicine, The National University of Malaysia, Cheras, 56000 Kuala Lumpur, Malaysia; 2 Department of Pathology, UKM Medical Centre, Faculty of Medicine, The National University of Malaysia, Cheras, 56000 Kuala Lumpur, Malaysia

**Keywords:** Thrombotic thrombocytopenia purpura, ADAMTS13 deficiency, Children

## Abstract

**Background:**

Acquired thrombotic thrombocytopenia purpura is very rarely encountered in children. It is often misdiagnosed initially when the condition is not inherited.

**Case presentation:**

We describe a 3-year-old Malay boy who presented with simple febrile seizure and had no neurological deficit, however, he was found to have microangiopathic hemolytic anemia, thrombocytopenia, and elevated serum lactate dehydrogenase. An ADAMTS13 assay results showed zero activities (0%), and markedly high level of ADAMTS13 inhibitor (93.15 U/mL) confirming the diagnosis of secondary thrombotic thrombocytopenia purpura. He received fresh frozen plasma infusions for 3 days and subsequently his platelet levels normalized. Serial ADAMTS13 assay results showed improvement. He was also given a short course of prednisolone after which the ADAMTS13 activity normalized (> 114%) at the end of prednisolone course.

**Conclusions:**

At presentation, acquired thrombotic thrombocytopenia purpura in a very young child is commonly misdiagnosed as other conditions like idiopathic thrombocytopenic purpura, Evans syndrome, atypical hemolytic-uremic syndrome, or malignancy. ADAMTS13 assay should be performed early when thrombotic thrombocytopenia purpura is suspected as this condition is associated with dire consequences.

## Background

Acquired thrombotic thrombocytopenia purpura (TTP) is very rare in children, and this condition is due to severe deficiency of disintegrin and metalloproteinase with a thrombospondin type 1 motif, member 13 (ADAMTS13) activity. TTP is characterized by microangiopathic hemolytic anemia (MAHA), thrombocytopenia, and elevated serum lactate dehydrogenase (LDH). Diagnosis of TTP is confirmed by the measurement of a serum ADAMTS13 activity level of < 10% (normal range, 40–130%) or undetectable in the acute phase [1–3]. ADAMTS13 is a family of 19 metalloprotease enzymes, and is responsible for cleaving the ultra-large von Willebrand factor (vWF) released from endothelial cells, hence, reducing the vWF multimeric size [2, 4, 5]. A functional deficiency of ADAMTS13 can cause accumulation of the ultra-large vWF in the plasma, thus forming platelet thrombi within the microcirculation. As a result, consumptive thrombocytopenia, mechanical hemolytic anemia, and multi-visceral ischemia can occur [2–4]. Congenital TTP is due to *ADAMTS13* gene mutation (Upshaw–Schulman syndrome) that usually presents with severe jaundice and thrombocytopenia in the neonatal period. Acquired TTP is frequently due to autoimmune formation of specific anti-ADAMTS13 autoantibodies, and anti-ADAMTS13 immunoglobulin G (IgG) should be investigated to confirm the diagnosis [2, 4].

The primary aim of this report is to highlight that acquired TTP should be suspected in a very young child who presents with febrile seizure without evidence of neurological findings, and has MAHA, thrombocytopenia, and elevated serum LDH. A high index of suspicion is required to diagnose this condition in children. Plasma infusion and corticosteroid treatment successfully induced complete remission in this patient despite not receiving standard treatment of plasmapheresis.

## Case presentation

Our patient was a 3-year-old Malay boy, who presented with fever of 2 days’ duration and an episode of generalized tonic-clonic seizure that lasted approximately 5 minutes. There was no up rolling of eye ball or drooling of saliva during the seizure. He had post-ictal drowsiness for 10 minutes. There were no other associated symptoms. Six weeks prior to the presentation he was brought to a general practitioner for fever and skin rashes over his face and upper limbs. He was treated with orally administered paracetamol and cefuroxime axetil. Subsequently, the fever resolved but the skin rashes persisted. On admission, he was diagnosed as having simple febrile seizure and eczema herpeticum. At 13 months of age he was diagnosed as having single gene deletion α-thalassemia trait (αα/−α4.2), and remained asymptomatic since diagnosis. His parents were non-consanguineous. His mother is 35-years old and has α-thalassemia trait. There was history of right ear infection a year before and that had resolved with treatment. He has history of allergy and intermittently was on orally administered desloratadine. There was no other significant past medical history, and he was not on any other medicine prior to the recent presentation. His immunization status was up to date, and developmentally he was normal. He lived with his parents who worked as police officers, and he has another healthy younger sibling of 5-months old. They lived in an apartment in a suburban area. He went to kindergarten when the parents worked.

**Fig. 1:**
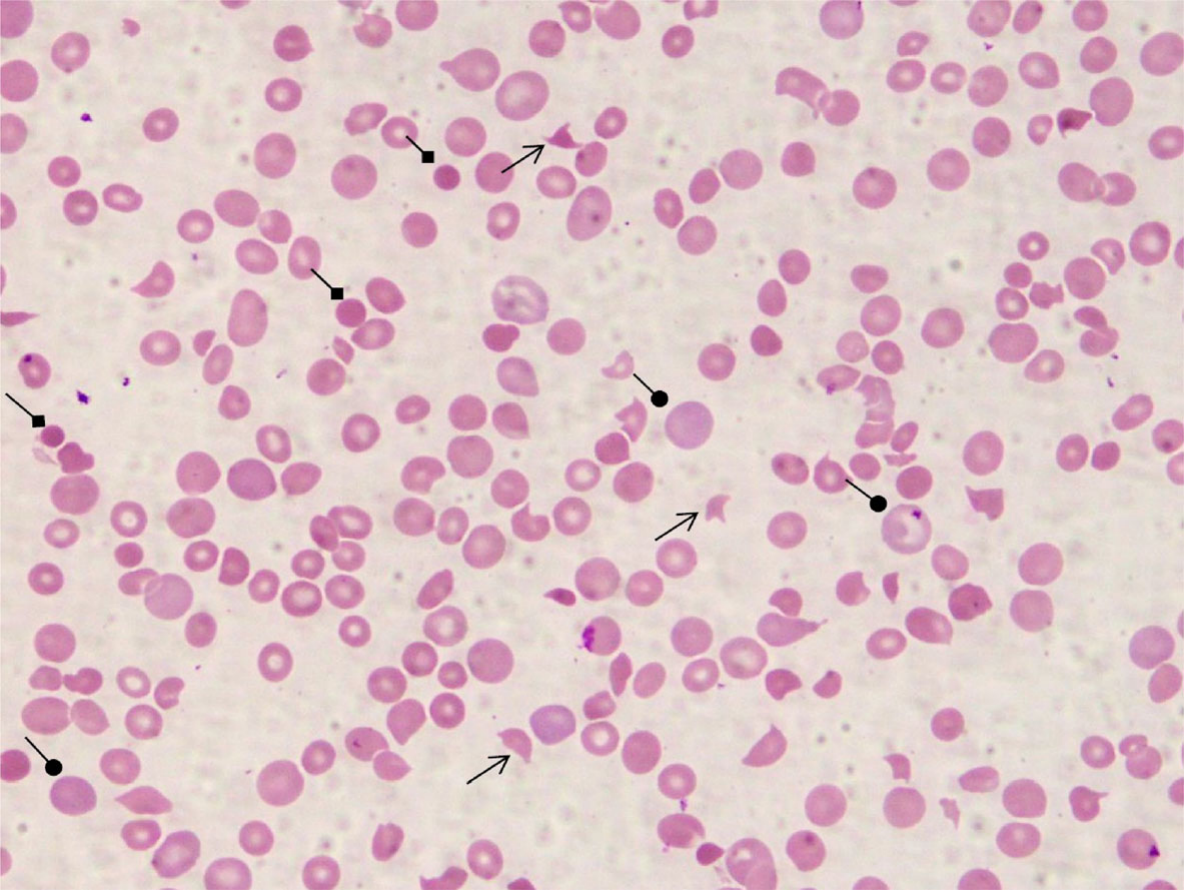
The peripheral blood film shows presence of fragmented red cells (*pointed-head arrow*), spherocytes (*diamond-head arrow*), increased polychromasia (*round-head arrow*), and thrombocytopenia

**Fig. 2:**
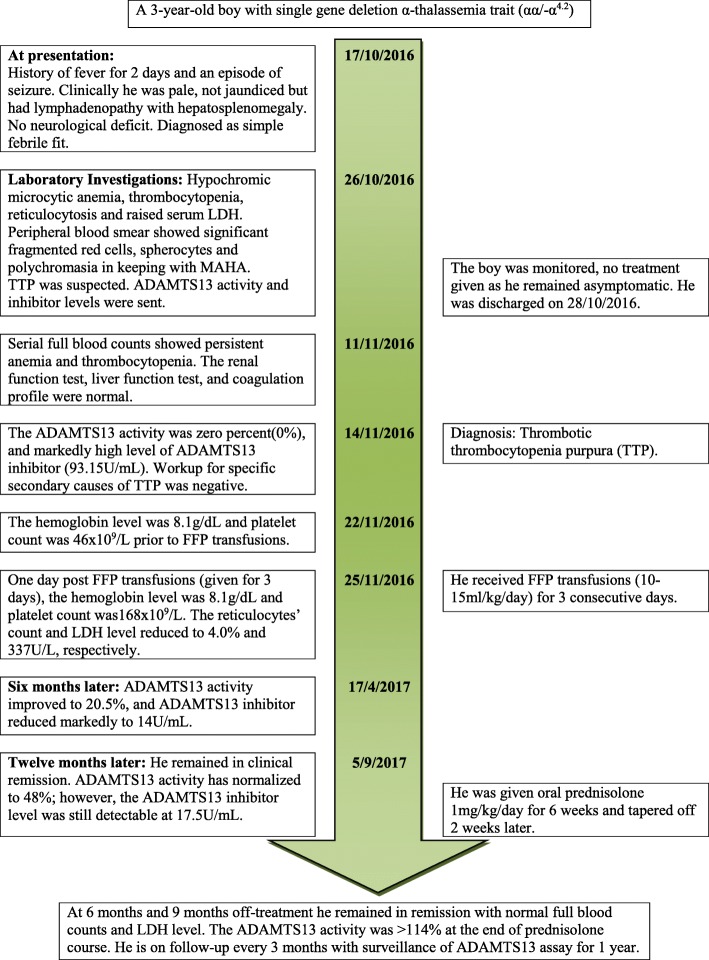
Timeline of illness. *ADAMTS13* a disintegrin and metalloproteinase with a thrombospondin type 1 motif, member 13, *FFP* fresh frozen plasma, *LDH* lactate dehydrogenase, *MAHA* microangiopathic hemolytic anemia, *TTP* thrombotic thrombocytopenia purpura

On admission, a physical examination revealed Glasgow Coma Scale of 15/15, blood pressure 90/46 mmHg, pulse rate 120/minute, and temperature 37.9 °C. He was febrile, pale, with no jaundice, and he had “shotty” cervical lymph nodes. The results of examinations of his throat, tonsils, and ears were normal. Some maculopapular rashes with scaly and crusted areas were noted on his left cheek, both arms and knees, and trunk area. There was hepatosplenomegaly of 4 cm and 3 cm, respectively. There were no bleeding tendencies or neurologic deficit noted. Laboratory findings showed hypochromic microcytic anemia, thrombocytopenia, reticulocytosis, and raised serum LDH. A peripheral blood smear showed significant fragmented red cells, spherocytes, and polychromasia in keeping with MAHA (Fig. 1). Serial investigation results showed persistent anemia and thrombocytopenia. His renal function test, liver function test, and coagulation profile were normal (Table 1). Direct and indirect Coombs tests were negative (Table 2). He was started on orally administered acyclovir for 10 days and received a course of cloxacillin for the rashes. TTP was suspected at this juncture. He was clinically asymptomatic, and was observed without any intervention prior to the results of ADAMTS13 assay. Subsequently, the ADAMTS13 assay results showed zero activities (0%) and markedly high level of ADAMTS13 inhibitor, 93.15 U/mL (negative < 12 U/mL; borderline 12–15 U/mL; positive > 15 U/mL) confirming the diagnosis of secondary TTP. Other laboratory investigations to identify specific secondary causes of TTP were negative: (i) viral studies on parvovirus, Epstein–Barr virus, and dengue virus; and (ii) blood culture and sensitivity. The screening for autoimmune disease showed anti-nuclear antibody positive and borderline double-stranded deoxyribonucleic acid (DNA), however, complement 3 and complement 4 levels were normal. He had no clinical findings to suggest connective tissue disease. He received fresh frozen plasma (FFP) transfusions (10–15 ml/kg per day) for 3 consecutive days. His hemoglobin (Hb) level was 8.1 g/dL and platelet count was 46 × 10^9^/L prior to the transfusions. Subsequently, the counts recovered. He had a Hb level of 8.1 g/dL and platelet count of 168 × 10^9^/L, a day after completed FFP transfusions. His reticulocytes count and LDH level reduced to 4.0% and 337 U/L, respectively. Six months later, the ADAMTS13 activity improved to 20.5%, and ADAMTS13 inhibitor reduced markedly to 14 U/mL. At 12 months, he remained in clinical remission, the ADAMTS13 activity had normalized to 48%; however, the ADAMTS13 inhibitor level was still detectable at 17.5 U/mL. He was given orally administered prednisolone 1 mg/kg per day for 6 weeks and it was tapered off 2 weeks later. A repeated ADAMTS13 activity at the end of the prednisolone course was > 114%. However, there was no result available about the inhibitor level. He is scheduled for follow-up every 3 months with surveillance of ADAMTS13 assay for 1 year. During clinic follow-up at 6 months after treatment, he remained in remission and his full blood counts were normal. A timeline for our patient is given in Fig. 2.

**Table 1:**
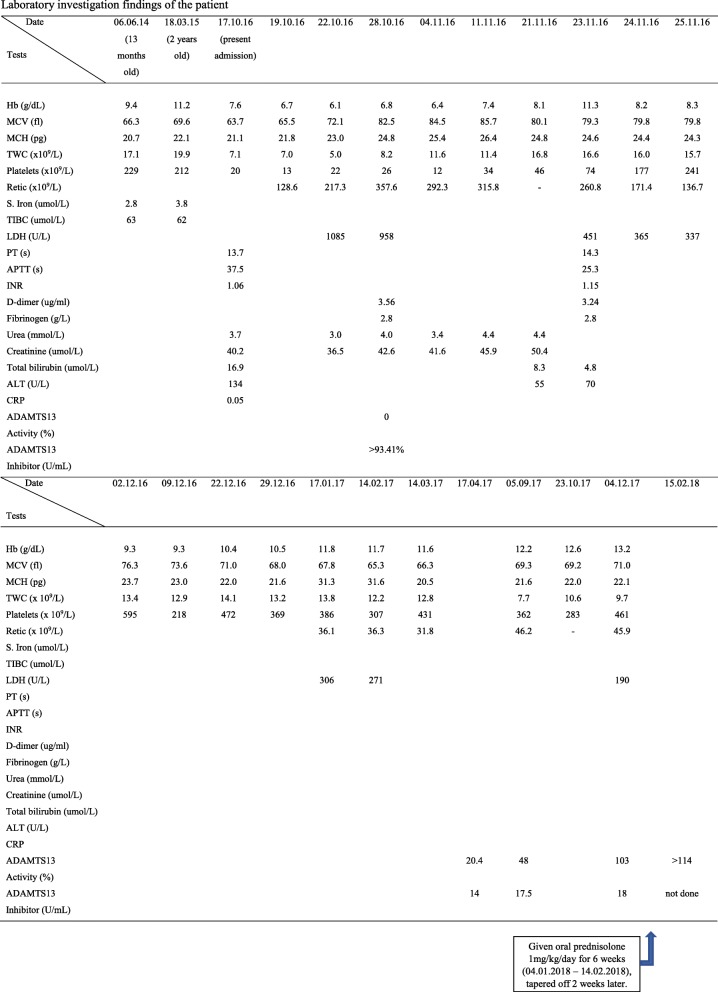
Laboratory investigation findings of the patient

*ADAMTS13* a disintegrin and metalloproteinase with a thrombospondin type 1 motif, member 13, *ALT* alanine aminotransferase, *APTT* activated partial thromboplastin time, *CRP* C-reactive protein, *Hb* hemoglobin, *INR* international normalized ratio, *LDH* lactate dehydrogenase, *MCH* mean corpuscular hemoglobin, *MCV* mean corpuscular volume, *PT* prothrombin time, *Retic*. reticulocyte count, *S. iron* serum iron, *TIBC* total iron-binding capacity, *TWC* total white cell

**Table 2:** Additional laboratory investigation findings of the patient

Investigations	17/10/16	20/10/16	11/11/16	14/11/16	14/02/17
Blood culture and sensitivity	No growth				
Dengue NS1 antigen	Non-reactive				
Dengue IgM	Negative				
Dengue IgG	Negative				
*Mycoplasma pneumoniae* IgM	Negative				
*Mycoplasma pneumoniae* IgG	1/80				
Epstein–Barr virus IgM	Negative				
Epstein–Barr virus IgG	Positive				
Parvovirus IgM	Negative				
Parvovirus IgG	Negative				
RPR/VDRL				Non-reactive	
HBsAg				Non-reactive	
Anti-HCV				Non-reactive	
HIV antigen and antibody				Non-reactive	
Complement 3 (mg/dL) (NR, 39–180)		109			136
Complement 4 (mg/dL) (NR, 10–40)		18.4			18.7
Anti-nuclear antibody (ELISA)		Positive			Positive
Anti-nuclear antibody (IF)		Positive (1/640) Pattern: speckled			Positive (1/640) Pattern: speckled
Anti-double-stranded DNA (ELISA)		Negative			Borderline
Immunoglobulin A (mg/dL) (NR, 70–400)		187			
Immunoglobulin M (mg/dL) (NR, 40–230)		87.2			
Immunoglobulin G (mg/dL) (NR, 700–1600)		1600			
Direct Coombs test		Negative			
Indirect Coombs test		Negative			
Erythrocyte sedimentation rate (mm/hour)			115		
Prothrombin time (seconds) (NR, 11.9–14.0)			14.2		13.7
Thrombin time (seconds) (NR, 12–21)			16.7		16.1
Fibrinogen (g/L) (NR, 1.36–4.65)			2.58		2.60
PTT LA			48.5		42.4
DRVV screen (seconds) (NR, 28–46.3)			41.7		37.9
DRVV screen ratio			1.04		0.92
Anti-cardiolipin IgM (U/mL) (NR, 0–20)			3.9		
Anti-cardiolipin IgG (U/mL) (NR, 0–20)			9.6		
Anti-beta2 glycoprotein 1 IgM (U/mL) (NR, 0–20)			2.7		1.1
Anti-beta2 glycoprotein 1 IgG (U/mL) (NR, 0–20)			43.5		12.3
Nuclear antibodies					
SmD			Negative		
RNP-70 K			Negative		
RNP-A			Negative		
RNP-C			Negative		
SSA(Ro52)			Negative		
SSA(Ro60)			Negative		
SSB(La)			Negative		
Cenp-B			Negative		
Topo-1(Scl-70)			Negative		
Ribosomal P			Negative		
Histone			Negative		
SmB			Negative		
Jo-1(HRS)			Negative		

*Cenp-B* centromere protein B, *DRVV* dilute Russell’s viper venom test, *ELISA* enzyme-linked immunosorbent assay, *HBsAg* hepatitis B surface antigen, *HCV* hepatitis C virus, *HIV* human immunodeficiency virus, *IF* immunofluorescent, *Jo-1(HRS)* histidyl-tRNA synthetase, *NR* normal range, *NS1* nonstructural protein 1, *PTT LA* prothrombin time lupus anticoagulant, *RNP* ribonucleoprotein, *RPR/VDRL* rapid plasma reagin/Venereal Disease Research Laboratory, *Sm* Smith protein, *SSA(Ro)* Sjögren’s syndrome-related antigen A, *SSB(La)* Sjögren’s syndrome-related antigen B, *Topo-1(Scl-70)* topoisomerase 1

## Discussion

Our case report describes a very young boy with acquired TTP who presented with febrile seizure during an infection, and had no clinical evidence of neurological deficit at presentation. Laboratory investigations revealed MAHA, thrombocytopenia, elevated serum, and LDH, and an ADAMTS13 assay confirmed secondary TTP. He recovered with treatment of plasma infusion and corticosteroid, and did not receive the standard treatment of plasmapheresis.

In children, acquired TTP is very uncommon and can be life-threatening [1, 3]. TTP is diagnosed when the classic pentad of fever, MAHA, thrombocytopenia, neurologic manifestation, and elevated creatinine are present. However, not all signs are observed at initial presentation and the disease varies in severity [1–3]. The Oklahoma TTP-HUS (hemolytic-uremic syndrome) Registry reported that the incidence rate of TTP in children was 2.24 × 10^6^ children/year [1]; acquired TTP has an annual incidence rate of 0.09 × 10^6^ children/year [1]. A cohort study of the French national registry for thrombotic microangiopathy reported a prevalence of 1 case per 1 million children for childhood-onset acquired TTP [3]. Acquired TTP was reported to be more common among older children (9–17 years of age) and female gender [1, 3]. However, we report here a very young boy with acquired TTP following an episode of febrile seizure with skin infection. In children, acquired TTP is often misdiagnosed initially as idiopathic thrombocytopenic purpura, Evans syndrome, atypical HUS (aHUS), or malignancy. HUS and TTP share a common underlying pathological process of thrombotic microangiopathy with detection of schistocytes in the peripheral blood smear. HUS is defined as the triad of MAHA, thrombocytopenia, and acute kidney injury. In typical diarrhea-associated HUS, the clinical manifestation frequently follows a prodromal hemorrhagic enterocolitis. In aHUS, abdominal pain and diarrhea are usually absent and the presentations could mimic TTP. However, the distinction between aHUS and TTP can be made by performing molecular genetics [6]. Genetic or acquired aHUS is associated with mutation within the gene coding for regulatory components for the complement system. TTP is diagnosed by reduction in ADAMTS13 activity [7]. Delay in diagnosis of TTP in children could result in severe consequences. We did not find any secondary causes in our patient. However, one should be aware that this condition is sometimes associated with other clinical problems in children such as diabetic ketoacidosis, acute lymphocytic leukemia, or viral infections [8, 9]. In acquired TTP, antibodies toward ADAMTS13 may develop idiopathically or in association with another autoimmune condition or connective tissue disease [1, 2, 4]. Brunner *et al.* reported that 26% of 35 patients with TTP had systemic lupus erythematosus (SLE), and an additional 23% had incipient SLE [10]. Despite the negative findings of an autoimmune condition in our patient, regular monitoring is needed during follow-up. Some patients were reported to develop SLE or other autoimmune states after the diagnosis of TTP.

Plasma exchange is the mainstay of treatment for acquired TTP, to remove the autoantibody against ADAMTS13 and the ultra-large vWF, while replacing the missing protease in the patient [1, 2, 4, 11]. Recently, a study reported benefits of plasma transfusions in some patients with TTP when 70–80% of them achieved remission [12]. It is interesting to note that our patient’s condition resolved with FFP transfusions and steroid. However, plasma transfusions alone could be hampered by fluid overload and proteinuria in patients [11, 12]. Hence, in the setting where plasma exchange may not be immediately available to patients, plasma transfusions can be effective as a temporary measure [11, 12]. Recent developments in therapeutic options for TTP include the use of caplacizumab (an anti-vWF immunoglobulin) and recombinant ADAMTS13 [13–15]. In a life-threatening condition, clinical presentation and the triad findings are often sufficient to diagnose TTP and justify emergency treatment [2, 4]. At the same time, initial measurement of ADAMTS13 activity is vital and should be done to confirm the diagnosis [2, 4, 16]. In patients with very low ADAMTS13 level and high level of ADAMTS13 inhibitor, the risk of relapse was threefold greater than those with higher ADAMTS13 level and without inhibitor [17, 18]. Regular follow-up to monitor the clinical condition, platelet count, and LDH, and ADAMTS13 assay is crucial to detect relapse, and prompt treatment could avoid mortality [17–19].

## Conclusions

Acquired TTP is very rare among young children and often misdiagnosed at presentation. When a child presents with febrile seizure, MAHA, thrombocytopenia, and elevated LDH, TTP should be suspected. ADAMTS13 assay should be performed early as this condition is associated with dire consequences.

## Abbreviations

ADAMTS13: A disintegrin and metalloproteinase with a thrombospondin type 1 motif, member 13
aHUS: Atypical hemolytic-uremic syndrome
DNA: Deoxyribonucleic acid
FFP: Fresh frozen plasma
Hb: Hemoglobin
HUS: Hemolytic-uremic syndrome
IgG: Immunoglobulin G
LDH: Lactate dehydrogenase
MAHA: Microangiopathic hemolytic anemia
SLE: Systemic lupus erythematosus
TTP: Thrombotic thrombocytopenia purpura
vWF: von Willebrand factor
